# 2015 Estimation of Hospitals Safety from Disasters in I.R.Iran: The Results from the Assessment of 421 Hospitals

**DOI:** 10.1371/journal.pone.0161542

**Published:** 2016-09-07

**Authors:** Ali Ardalan, Maryam Kandi Keleh, Amin Saberinia, Davoud Khorasani-Zavareh, Hamidreza Khankeh, Jafar Miadfar, Samaneh Maleknia, Atieh Mobini, Sara Mehranamin

**Affiliations:** 1 Department of Disaster and Emergency Health, National Institute of Health Research, Tehran University of Medical Sciences, Tehran, Iran; 2 Department of Disaster Public Health, School of Public Health, Tehran University of Medical Sciences, Tehran, Iran; 3 Harvard Humanitarian Initiative, Harvard University, Cambridge, MA, United States of America; 4 Disaster and Emergency Management Center, Ministry of Health and Medical Education, Tehran, Iran; 5 Department of Health in Disaster and Emergency, School of HSE, Shahid Beheshti University of Medical Sciences, Tehran, Iran; 6 Department of Clinical Science and Education, Karolinska Institute, Stockholm, Södersjukhuset, Sweden; University of Manchester, UNITED KINGDOM

## Abstract

**Background and Objective:**

Iran’s health system has developed a Farsi edition of the Hospital Safety Index (HSI) and has integrated the related assessment program into the health information system. This article presents the results of the 2015 estimation of hospital safety from disasters in I.R.Iran using HSI.

**Methods:**

We analyzed data from 421 hospitals that had submitted a complete HSI assessment form on the Ministry of Health and Medical Education Portal System. Data collection was based on the self-assessments of the hospital disaster committees. HSI includes 145 items categorized in three components including, structural, non-structural and functional capacity. For each item, safety status was categorized into three levels: not safe (0), average safety (1) and high safety (2). A normalized scoring scheme on a 100-point scale was developed. Hospitals were classified to three safety classes according to their normalized total score: low (≤34.0), average (34.01–66.0) and high (>66.0).

**Results:**

The average score of all safety components were 43.0 out of 100 (± 11.0). Eighty-two hospitals (19.4%) were classified as not safe, and 339 hospitals (80.6%) were classified in the average safety category. No hospital was placed in the high safety category. Average safety scores were 41.0, 47.0, and 42.0 for functional capacity, non-structural safety, and structural safety respectively. The average safety score increased between 2012 and 2015, from 34.0 to 43.0.

**Conclusions:**

Hospital safety in the event of disasters has improved in Iran in recent years and more hospitals have joined the HSI program. This is a result of continuous efforts invested in capacity building programs and promotion of the 2012 HSI estimation. The HSI should be maintained to monitor the progress of Iran’s health system in regards to hospital safety in the case of disasters. It is recommended that WHO continue advocacy of HSI, establish a HSI monitoring system, and add it to country profiles on WHO website.

## Introduction

Following the 2008–2009 International Day of Disaster Reduction (IDDR) [1], Iran launched a national campaign on Hospitals Safe from Disasters, starting with a conference at Tehran University of Medical Sciences (TUMS) in October 2009. The conference was held in collaboration with the Ministry of Health and the Medical Education (MoHME); World Health Organization (WHO), Tehran Office; and United Nations International Strategy for Disaster Reduction (UNISDR) Tehran Office. This effort led to the ultimate integration of the Hospital Safety Index (HSI) into the health information system of I.R.Iran [2].

HSI is a tool developed by the WHO and recommended as a rapid, reliable and cost-effective diagnostic instrument that addresses the structural safety, non-structural safety and functional capacity of a hospital in 145 areas [3]. It has an all hazards approach with a special focus on earthquake in the section of structural component. The index was originated from Pan American Health Organization (PAHO) and the Latin America countries. Accordingly, following the global campaign of hospitals safe from disasters, it was extensively applied in other regions including Europe [4,5]. The HSI was translated to Farsi and adapted to Iran’s context as well. The adapted tool was named Farsi Hospital Safety Index (FHSI). Accordingly, the MoHME called upon Iranian hospitals to assess their safety from disasters using the FHSI. In 2012, about one-fourth of Iranian hospitals, i.e., 224 hospitals, joined this program, completed the assessment, and reported back to MoHME. The methods and results of this assessment have been previously published [6].

**Fig 1:**
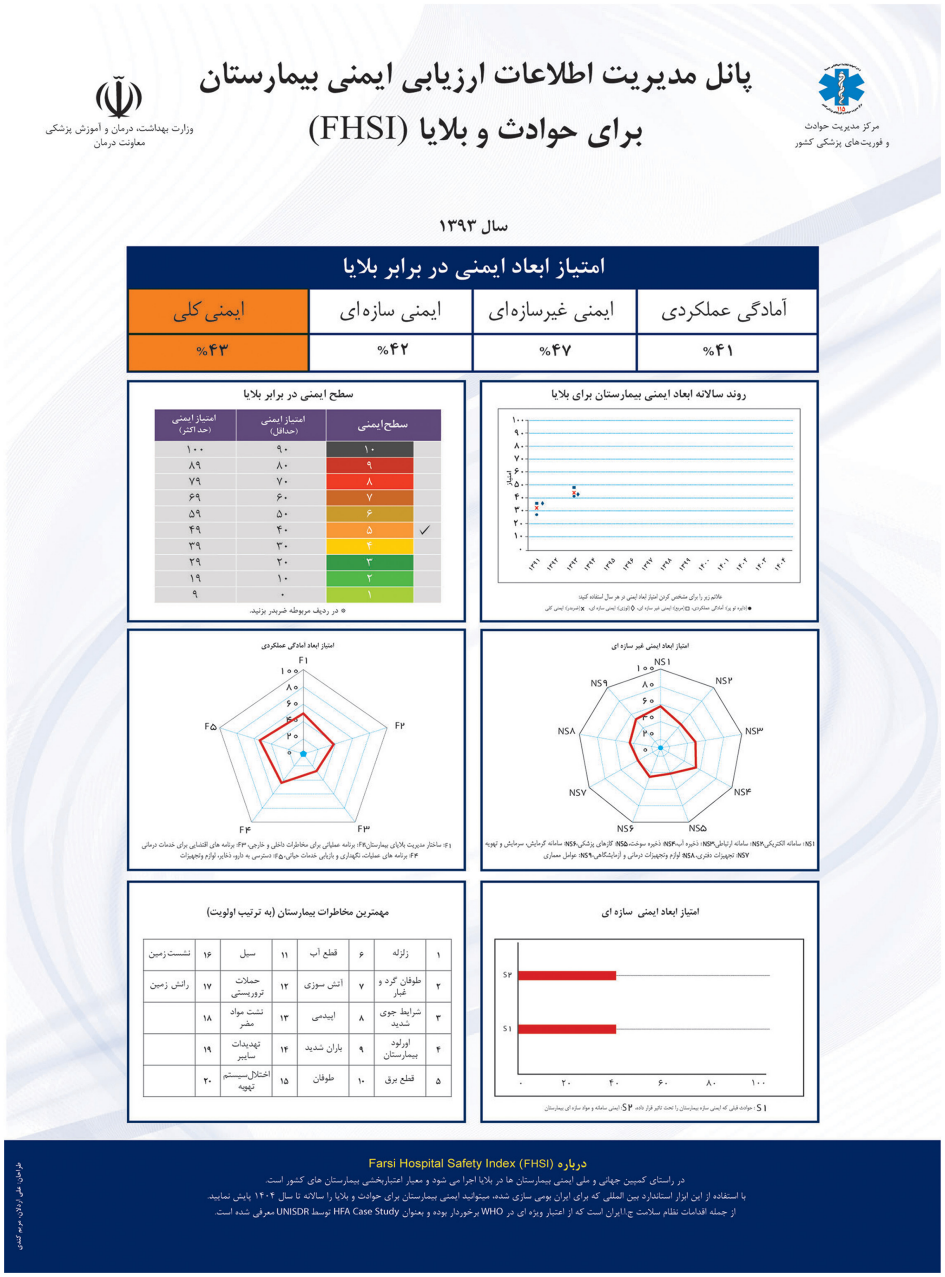
FSHI dashboard posted in office of Health Deputy Minister.

The findings of the 2012 assessment were applied to advocate for hospital safety in the event of disasters amongst policy makers, and encouraged them to support the sustainability of the program. As the result, national and provincial capacity building programs were carried out and the 2015 edition of Hospitals Accreditation Protocol included the FHSI as criteria. Furthermore, a FHSI dashboard was developed and endorsed by MoHME. This dashboard summarizes the results of FHSI from an administrative hospital level to the national level. It should be posted in the offices of hospital managers and health authorities. Fig 1 shows the FSHI dashboard that is posted in office of Health Deputy Minister.

This article presents the results of the FHSI conducted in 421 Iranian hospitals in 2015, compared to the 2012 results. The findings can be applied in policy development in the health system of I.R.Iran.

## Methods

The methods for adapting the HSI to Iranian context was described in details previously [6]. In summary, the adaptation was performed by a multidisciplinary group of experts from disaster management, medical sciences, architecture and engineering. The adaptation process also included translation to the Farsi language, field-testing, face and content validation, and developing an analysis plan.

For the year of 2015, we analyzed the FHSI data that were collected in 2015 and were available on the MoHME portal system. After checking the data, 32 hospitals were determined to have incomplete submissions and were excluded. Finally, 421 hospitals were considered for the analysis. The hospitals with incomplete submission were all public hospitals affiliated to MoHME. However, no other data was available on the portal system about these hospitals.

According to the FHSI guideline, hospital disaster committees (HDC) were responsible for assessment coordination, data collection, and data entry in the MoHME Portal System. The assessment teams included three to five members including doctors, nurses, technicians, or engineers from the hospital maintenance office. Self-assessment was the primary approach for data collection. Data was entered into an Excel spreadsheet and uploaded to the portal system of MoHME. To answer queries from the data collection team, two officers were available within the MoHME during working hours.

Table 1 shows the safety components and corresponding elements in the FHSI. The safety assessment included three components covering structural, non-structural, and functional capacity. The evaluators also were expected to fill in the forms related to the hospital’s general information and hazards identification. All evaluators also passed a comprehensive three days course for data collection and data entry in FHSI.

**Table 1:** Components of hospital safety in Farsi Hospital Safety Index.

Safety component	Safety element		Number of items
Structural	S1	Previous events affecting the safety of hospital buildings	3
	S2	Safety of structural systems and materials used in buildings	10
Non-structural	NS1	Electrical system	8
	NS2	Telecommunications system	7
	NS3	Water supply	5
	NS4	Fuel storage	4
	NS5	Medical gases	7
	NS6	Heating, ventilation, and air-conditioning (HVAC) systems in critical areas	7
	NS7	Office, storeroom furnishings, and equipment (fixed and movable) including computers, printers, etc.	3
	NS8	Medical/laboratory equipment and supplies used for diagnosis and treatment	12
	NS9	Architectural elements	18
Functional capacity	F1	Organization of Hospital Disaster Committee and Emergency Operations Center	11
	F2	Operational plan for internal or external disasters	24
	F3	Contingency plans for medical treatment in disasters	8
	F4	Plans for the operation, preventive maintenance, and restoration of critical services	8
	F5	Availability of medicines, supplies, instruments, and other equipment for use in emergencies	10
Total			145

To analyze the data, the safety status of each item was categorized to three levels: not safe, average safety and high safety. We assigned scores of 0, 1 and 2 to each category, respectively. Equal weight was given to all safety components and corresponding elements. A raw score was tallied by a simple sum of all the item scores. Finally, all scores were normalized on a 100-point scale. To ease interpretation, all scores were rounded to the nearest round number. Furthermore, hospitals were classified into three safety classes according to the normalized total scores as follows: low (≤34.0), average (34.01–66.0) and high (>66.0).

The safety scores were compared based on hospital affiliation (MoHME, Social Welfare Organization (SWO), private, charity, military and oil company), hospital function (general *vs*. specialized) and hospital size (≤100 *vs*. >100 beds). Descriptive analysis, one-way ANOVA, independent t-test and chi-square were the statistical tests applied, where appropriate SPSS 19.0 was used for statistical analysis. P<0.05 was considered as statistically significant.

## Results

In 2015 in total, 421 Iranian hospitals submitted complete FHSI forms to the MoHME, with a rough 42% response rate. Table 2 presents the characteristics of the hospitals, along with the results of their safety assessment.

**Table 2:** Average safety score of functional capacity for disasters in 421 of Iran’s hospitals, 2015.

Hospital type	n	%	Functional capacity	Non-structural safety	Structural safety	Total safety
**Hospital affiliation**						
MOHME*	286	67.9	39 (16)	46 (15)	42 (18)	42 (11)
SWO†	35	8.3	47 (14)	48 (10)	50 (15)	48 (7)
Private	71	16.9	48 (12)	50 (14)	36 (17)	45 (11)
Charity	16	3.8	39 (21)	46 (23)	41 (26)	42 (15)
Military	11	2.6	64 (9)	48 (24)	28 (19)	47 (10)
Oil company	2	0.5	20 (3)	50 (1)	44 (8)	32 (4)
*P value*			*<0*.*001*	*<0*.*001*	*0*.*053*	*0*.*078*
**Hospital function**						
General	295	70.1	41 (16)	48 (14)	45 (17)	45 (10)
Specialized	126	29.9	41 (17)	43 (17)	34 (19)	40 (13)
*P value*			*<0*.*001*	*0*.*006*	*<0*.*001*	*<0*.*001*
**Hospital size**						
≤100 beds	174	41.2	39 (16)	49 (13)	44 (19)	44 (11)
>100 beds	227	58.8	43 (16)	45 (16)	40 (18)	43 (11)
*P value*			*<0*.*001*	*0*.*036*	*0*.*086*	*<0*.*001*
**All hospitals**	421	100	41 (16)	47 (15)	42 (18)	43 (11)

*MOH&ME: Ministry of Health and Medical Education

^†^SWO: Social Welfare Organization

Eighty-two hospitals (19.4%) were classified as low safety, and 339 hospitals (80.6%) were classified as having average safety. No hospital was placed in the high safety category. The average score of all safety components was 43.0 out of 100 (± 11.0).

**Fig 2:**
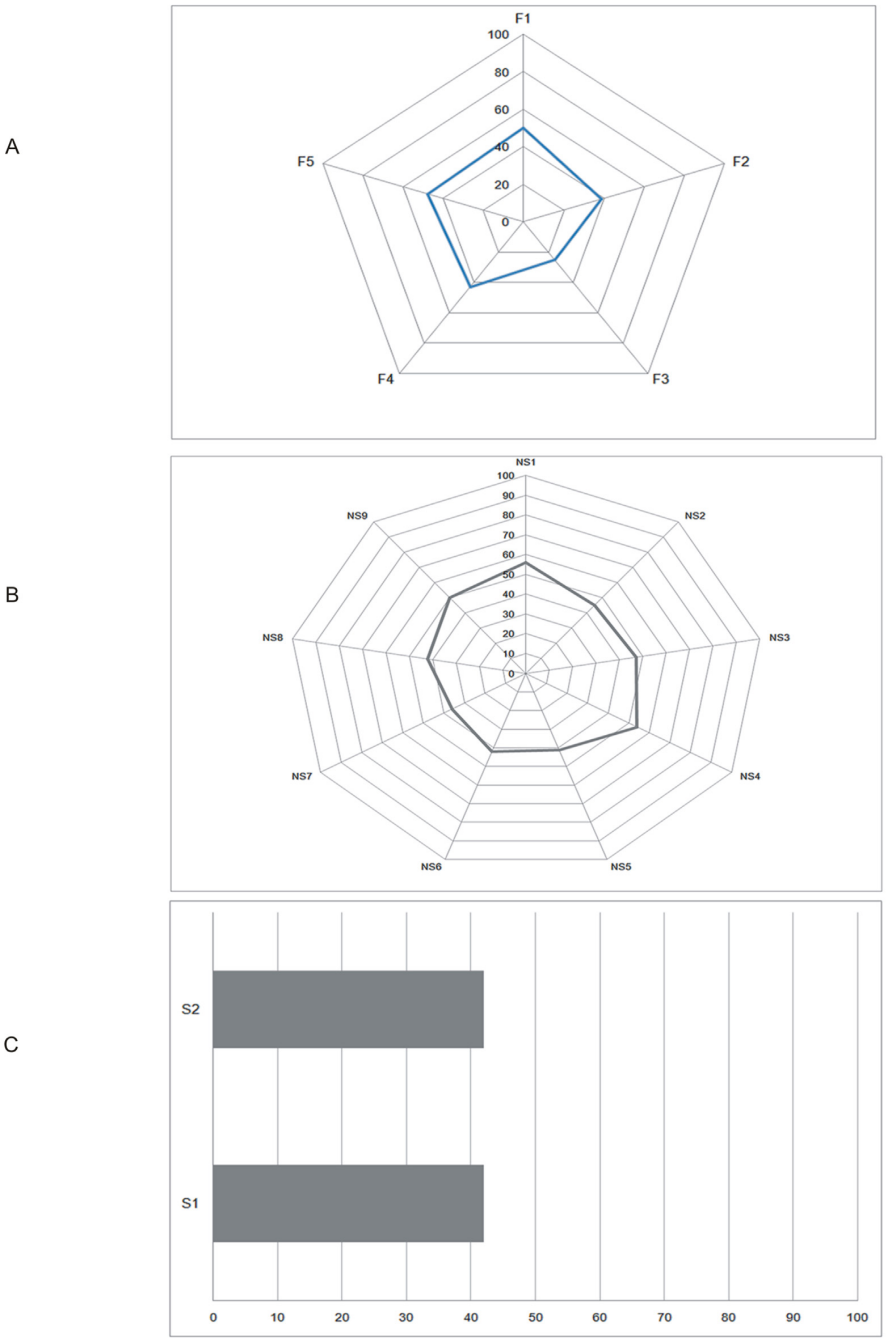
Disaster safety score in terms of A) functional capacity, B) non-structural safety, and C) structural safety in 421 of Iran’s hospitals, 2015.

Fig 2A, 2B and 2C, visualize the safety scores of hospitals in terms of various elements of functional capacity, non-structural safety and structural safety.

Regarding the various elements of functional capacity, the average safety score ranged from 25.0 to 50.0 for the “contingency plans for medical treatment in disasters” and the “organization of hospital disaster committee and emergency operations center” respectively. The average safety score was 41.0 out of 100 (±16.0 for all elements of functional capacity).

In regards to the non-structural elements, the average safety score ranged from 36.0 to 56.0 for the “office and storeroom furnishings and equipment” and the “electrical system” respectively. The average safety score was 47.0 out of 100 (±15.0) for all elements within the non-structural component.

The average safety scores for both elements of structural safety was 42.0. The average safety score out of 100 was 42.0 (±18.0 for all elements within the structural component).

Table 2 presents the results of the safety assessment with consideration to safety components and type of hospital. The functional preparedness of military hospitals was significantly higher than others, while the lowest preparedness was observed in hospitals affiliated with the Oil Company. Functional preparedness was also higher in larger, rather than smaller hospitals.

The highest non-structural safety was observed in private and oil company hospitals as opposed to other hospitals; general rather than specialized hospitals; and larger hospitals rather than smaller hospitals. Structural safety was reported to be higher in the SWO hospitals than in general hospitals and hospitals with ≤100 beds.

The total safety score, in descending order, was found to be: SWO, military, private, MoHME, charity, and oil company hospitals. General hospitals and hospital with ≤100 beds showed higher levels of total safety.

The following 10 hazards were reported by the hospitals as hazards with the highest importance: Earthquake 71%, extreme temperature 64%, dust storm 59%, hospital overload 48%, power outage 48%, and water cut 46%, fire 45%, torrential rains 45%, storms 43% and landslides 39%.

**Fig 3:**
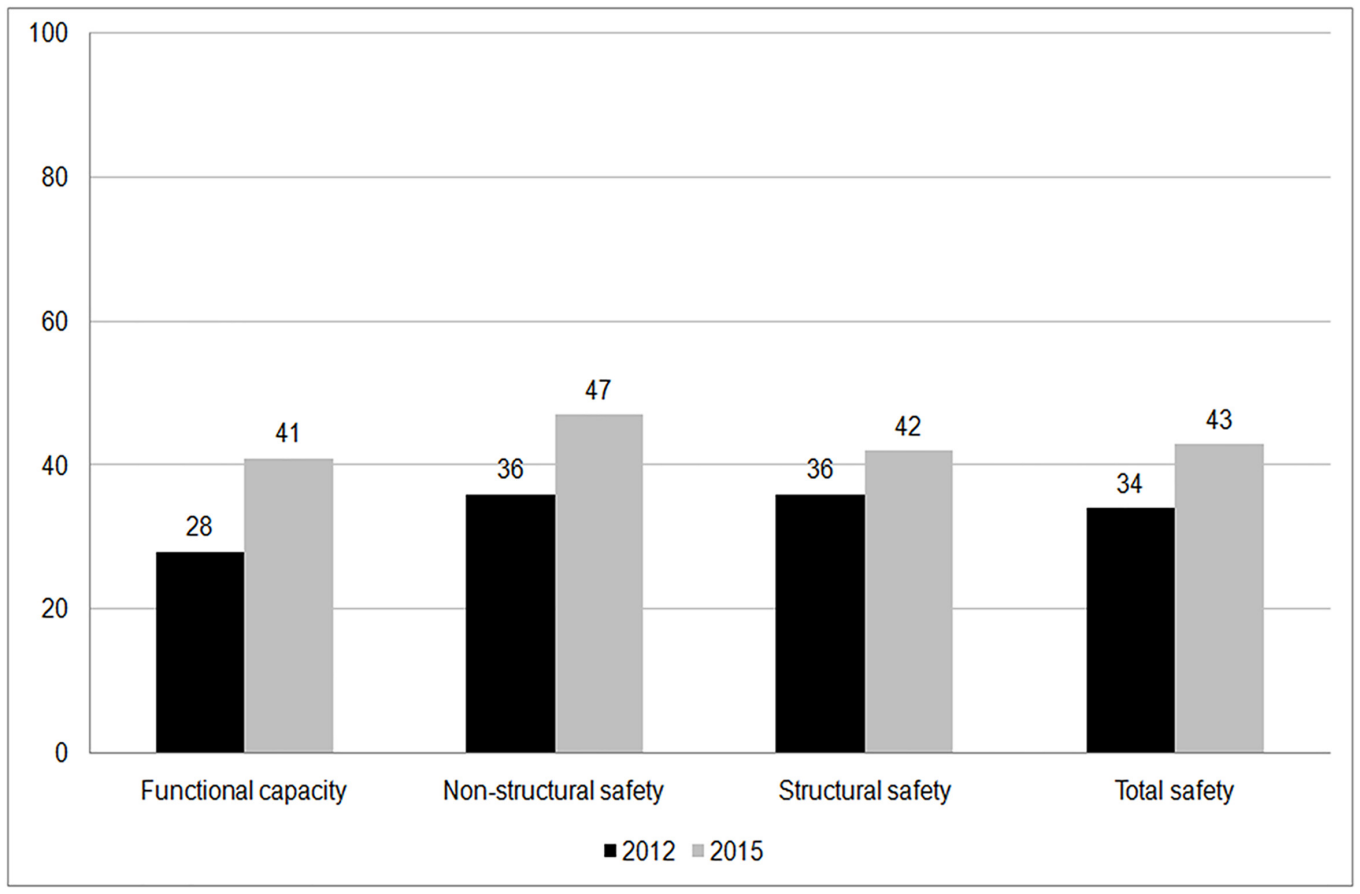
Trend of hospital disaster safety in I.R.Iran from 2012 to 2015, based on Farsi Hospital Safety Index.

Fig 3 compares the FHSI scores between 2012 and 2015. An increasing trend of all safety components can be seen. The average total safety score increased from 34.0 to 43.0 out of 100. The largest difference was seen in functional capacity.

## Discussion

Our findings showed that the total disaster safety of Iran’s hospitals was at 43% in 2015. It was 41% for functional capacity, 47% for non-structural safety and 43% for structural safety. About 20% of hospitals were found to be low in safety and the rest were determined as moderately safe. Compared to statistics from 2012, in addition to the additional hospitals that participated in the program in recent years, the total safety has also improved.

The second round of FHSI results present strong evidence regarding program sustainability in Iran. Furthermore, we expect higher participation of hospitals over the coming years, as hospital accreditation protocol requires the FHSI to be conducted. Hospitals should follow the protocol in order to renew their licenses. HSI has become a popular tool in different regions [7–8] and a well-established program in the WHO. The second edition of HSI [9] was released in early 2015 during the 3^rd^ World Conference on Disaster Risk Reduction in Sendai, Japan. In the second edition while the integrity of original tool has been maintained, some changes have been made. Examples, beside adding or removing items, include giving greater emphasis to security, staff availability, fire protection and suppression systems for internal fires, maintenance of critical systems, and the system for coordination of emergency operations in the hospital. Furthermore, the new edition addresses all types of hazards which may affect the safety of the hospital or lead to an emergency or disaster to which the hospital will need to be prepared to respond [9].

The FHSI team has already translated the new edition and tested it in pilot hospitals. It is expected to be released in 2016 and will be used for the next round of assessments in Iran.

The Sendai Framework for Disaster Risk Reduction (SFDRR) has highlighted the importance of safety of hospitals. It has explicitly emphasized the resilience of new and existing infrastructure, including hospitals to ensure that they remain safe, effective, and operational during and after disasters in order to provide life-saving and essential services [10]. It has also referred to the global campaign on safe hospitals as an example of interventions that needs to be accomplished. We believe that the best support from the SFDRR is the showcase of tangible results from the DRR program at the national and local level. The FHSI program of Iran can be used as a case study in this regard, as it was in the Hyogo Framework for Action (HFA) [2].

HSI helps countries monitor their progress in safety of hospitals, as it has done in Iran. Additionally, it is an educational tool that enhances knowledge of hospitals on disaster preparedness and safety. HSI can be considered as an indicator in monitoring the SFDRR targets and goals. HSI can also be applied for community risk assessments, as it has been used in a Vulnerability and Risk Assessment and Mapping (VRAM) project in the Kerman province of Iran. Furthermore, it is being used for a community risk assessment project in the capital city of Iran, Tehran.

The FHSI, as a screening tool, provides baseline information on hospital safety status for disasters in Iran. It can be completed by more detailed vulnerability and preparedness assessments. However, the key to the success of this program is repeating the FHSI assessment on a regular basis to evaluate the effectiveness of intervention programs over time.

There is the potential of selection bias in our findings, because of the non-participant hospitals. No data was available at the time of analysis about characteristics of the non-participant hospitals. Our future goal is to expand the FHSI program to all hospitals in the nation- the accreditation protocol will be helpful in this regard.

The self-assessment approach stands as both the strength and weakness of Iran’s FHSI program. It is a strength for two reasons: 1) it has made the program feasible to be expanded throughout the country in a short period of time with minimum costs, compared to the cost that is needed for external evaluation of about 1,000 hospitals, and 2) this approach serves as a self-learning method for hospitals. We speculate the former has been an effective factor on improving FHSI scores during two rounds of assessment. On the other hand, self-assessment can be a source for potential information bias. Research projects are necessary to estimate the amount of bias, employing correction factors in order to correct the measurements completed within the self-assessments.

We observed an improvement in the average FHSI scores from 2012 to 2015. This improvement is largely due to enhanced awareness within hospitals, training workshops on the FHSI and hospital disaster planning, disaster drills, and further developments that took place during this time period. Anecdotal evidence shows that hospitals are investing more and more on their physical safety, including non-structural safety and structural retrofitting. MoHME is working on a monitoring system to collect, analyze, and report interventional measures on hospital safety from disasters in the country.

Our findings revealed some statistically significant differences in the FHSI score among different types of hospitals. However, the most meaningful difference was in the improved functional preparedness of military hospitals, as expected. The remaining differences were not practically meaningful. In all, Iran’s health system must focus on the functional readiness of small hospitals (≤100 beds), and the physical safety of large hospitals (>100 beds).

Classification of the hospital safety in low, average, and high levels helps the health system of Iran prioritize hospital targets. However, the role of a hospital in the emergency plan of the designated area needs to be taken into consideration. Hospitals with low and average safety levels that are integral in Iran’s emergency management are highly recommended to enlist in immediate intervention programs.

Despite improvement in the safety of Iranian hospitals, there are still concerns about new hospitals that are under construction. We witnessed the destruction of newly constructed hospitals in the aftermaths of East Azarbaijan and Bushehr earthquakes. Improved supervision of the implementation of safety codes is required.

According to the roadmap of Iran’s health system for disaster risk management [11], the safety of health facilities in the event of a disaster is a national priority. This requires multi-disciplinary collaboration and commitment from high-level authorities. For example, the establishment of a national committee including the MoHME, SWO, military, Islamic Parliament, Ministry of Road and Urban Construction, private sector, Hospitals Accreditation and Supervision Center, Hospital Management Center, Physical Resources and Construction Office, and Disaster and Emergency Management Center is a critical step towards ensuring the safety of hospitals in the event of disasters.

The safety assessment of hospitals can help decision makers to prioritize the resources. The information obtained from the assessments can be used in design and construction of new hospitals, and in vulnerability reduction or capacity enhancement of existing hospitals [4]. Radovic V et al [4], Rockenschaub G et al [5], and Ingrassia PL et al [12] provided examples about usefulness and utilization of the hospital safety assessment in improving the process of decision making in European countries. Since the global campaign in 2008–2009, the WHO has extensively worked on advocacy of the hospital safety from disasters, but the HSI itself has not been the focus of advocacy programs. Authors believe that WHO should continue advocacy of the HSI worldwide, establish a monitoring system for this purpose, and include the HSI related indicators when publishing countries profile on WHO website.
